# Bilateral simultaneous total knee arthroplasty with and without patellar resurfacing. A prospective single surgeon series with a minimum follow-up of 7 years

**DOI:** 10.1186/s43019-024-00225-6

**Published:** 2024-05-29

**Authors:** Leonel Perez Alamino, German Garabano, Cesar Ángel Pesciallo, Hernán Del Sel

**Affiliations:** Department of Orthopaedics and Traumatology, British Hospital of Buenos Aires, Perdriel 74, C1280 AEB Buenos Aires, Argentina

**Keywords:** Total knee arthroplasty, Joint replacement, Patellar resurfacing, Knee osteoarthritis

## Abstract

**Background:**

Total knee arthroplasty (TKA) is the most effective treatment for end-stage adult knee osteoarthritis, but it has been reported that patient satisfaction may vary. A malfunction of the patellofemoral joint may produce anterior knee pain (AKP) for several reasons. While some surgeons systematically resurface the patella despite the risk of potential complications such as fracture, loosening, or wear of the patella, others prefer to preserve it to reduce AKP and revision rates. This study aimed to evaluate whether patellar resurfacing had better clinical and functional outcomes, complications, and revision rates in patients undergoing simultaneous bilateral total knee arthroplasty.

**Methods:**

We conducted a prospective cohort study, including patients who underwent bilateral simultaneous TKA in which the patella was replaced in one knee and preserved in the other, with a minimum follow-up of 7 years. We assessed clinical and functional outcomes with the Knee Society Score (KSS) and Visual Analogue Scale (VAS); complications and revision rates were also registered.

**Results:**

The final series consisted of 43 patients with 86 knee arthroplasties. After a mean of 7.6 years of follow-up, no significant differences were found regarding KSS (clinical: 82.8 ± 7.4 versus 83.2 ± 3.4, *p* = 0.92; functional 89.1 ± 8.2: versus 90.4 ± 6.8; *p* = 0.99), VAS (2.0 ± 0.9 versus 1.8 ± 1.0; *p* = 0.84), complications (10.5% versus 8.1%; *p* = 0.57), or revision rates (2.3% versus 2.3%; *p* = 0.99) when comparing patellar resurfacing versus retention.

**Conclusion:**

In the context of total knee arthroplasty, patellar replacement did not demonstrate statistically significant differences concerning patellar retention in clinical nor functional outcomes, AKP, complications, or revision rates after a minimum of 7 years of follow-up.

## Introduction

Total knee arthroplasty (TKA) is the most effective treatment for end-stage adult knee osteoarthritis (OA) [1–3], but it has been reported that patient satisfaction may vary [4]. Anterior knee pain (AKP) is a commonly cited reason for failure, although its cause is unclear [5, 6]. Malfunction of the patellofemoral joint (PFJ) may certainly produce AKP, maltracking, overstuffing, and wear, but it can also appear with synovial plicae, tendonitis, soft tissue scarring, and neuromas. In several cases, the origin of AKP will remain undiagnosed [7, 8].

Previous designs of femoral implants did not consider left or right patellar tracking, and the designers suggested patellar resurfacing as an integral part of the procedure [9]. With the emergence of patellar-friendly femoral designs, [10] some surgeons continued to resurface the patella in all cases [11–13] others chose to do it on an individual basis [14], and others chose not to resurface any patella regardless of the degree of affection of the PFJ [15, 16].

The literature abounds with prospective randomized trials and meta-analyses comparing all three possibilities, and despite this, the advisability of resurfacing the patella remains controversial and inconclusive [17, 18]. Those favoring patellar retention argue that this may minimize the potential complications of patellar fracture, mechanical loosening of the patellar button, and polyethylene wear [19]. A maltracking non-resurfaced patella will certainly increase patellofemoral arthritis, while a resurfaced patella will increase the amount of polyethylene debris, causing osteolysis and ultimate implant loosening [20, 21].

The purpose of this study was to evaluate whether patellar resurfacing had an impact on clinical and functional outcomes, postoperative AKP, and revision rates in patients undergoing simultaneous bilateral TKA. In this series, regardless of native patellar osteoarthritis, one patella was resurfaced and the other one underwent a patelloplasty with resection of peripheral osteophytes.

## Materials and methods

With the approval of the Institutional Review Board (protocol number 9956), the senior author (H.D.S.) initiated a prospective randomized data registry of patients operated consecutively of bilateral simultaneous TKA on a high-volume TKA center between June 2005 and December 2016. Simultaneous bilateral knee replacement was indicated in patients with severe osteoarthritis, with persistent pain despite medical treatment (physical therapy, non-steroid anti-inflammatory drugs, weight loss) for at least 3 months. General condition was previously assessed by cardiologist, anesthesiologist and clinical medical specialist. Inclusion criteria were of patients over 18 years with a diagnosis of end-stage bilateral tricompartmental knee OA, regardless of the degree of varus or valgus angular deformity in whom the patella was resurfaced in one knee and non-resurfaced in the other, with a minimum follow-up of 7 years. Patients with a history of previous knee surgery such as osteotomies, fractures, extensor realignment, anterior cruciate ligament reconstruction with bone-tendon-bone technique, and those with rheumatoid arthritis were excluded.

At the beginning of the study, we designed a list of cases numbered consecutively to randomize the patients and assign which patella to replace and which not to replace. Patients with odd case numbers (first, third, fifth) were operated first on the right knee and then on the left. Meanwhile, cases with even numbers (second, fourth, sixth, etc.) were operated on in reverse order (left knee first and then the right). In all cases, the patellar replacement was performed in the second operated knee, never in the first one. During this period, 46 bilateral simultaneous bilateral TKA were performed. One patient died before completing the minimum follow-up, and two patients had both patellae replaced (one because he had a history of previous surgeries and the other because she had rheumatoid arthritis) and were therefore excluded. The final series consisted of 43 patients with 86 TKA and a median follow-up of 7.6 years (range 7.0 to 10.2). The summary of preoperative characteristics is detailed in Table 1.

**Table 1:** Preoperative characteristics of patients included in the series

Variables	Overall	Resurfaced	Non-resurfaced	*p* value
Age _(mean, SD)_	68.3 ± 6.2	–	–	–
Male _(*n*, %)_	18 (41.9)	–	–	–
BMI _(mean, SD)_	28.1 ± 2.2	–	–	–
Kellgren-Lawrence _(*n*, %)_				
III	21 (24.4)	14 (32.5)	8 (18.6)	0.21
IV	65 (75.6)	29 (67.5)	35 (81.4)	
Preoperative Axis				
Varus	65 (75.6)			
Femorotibial angle _(mean, SD)_	7.1 ± 2.8	6.6 ± 2.5	7.4 ± 3.0	0.27
Valgus	21 (24.4)			
Femorotibial angle _(mean, SD)_	9.5 ± 4.1	10.6 ± 4.4	8.0 ± 3.4	0.18
Caton Deschamps _(mean, SD)_	0.97 ± 0.2	0.9 ± 0.2	1.0 ± 0.2	0.12
Preoperative ROM _(mean, SD)_				
Flexion	100.2 ± 11.4	99.4 ± 12.9	101.9 ± 9.1	0.75
Extension	8.2 ± 5.1	8.2 ± 4.1	8.7 ± 5.2	0.78
KSS _(mean, SD)_				
Clinical	47.2 ± 3.3	44.6 ± 4.8	45.5 ± 3.6	0.78
Functional	46.7 ± 5.0	45.3 ± 7.1	43.2 ± 7.8	0.84
VAS _(mean, SD)_	8.5 ± 1.1	8.4 ± 0.9	8.2 ± 1.0	0.99

BMI: body-mass index. ROM: range of motion. SD: standard deviation. KSS: Knee Society Score. VAS: Visual Analogue Scale

### Surgical technique

**Fig. 1:**
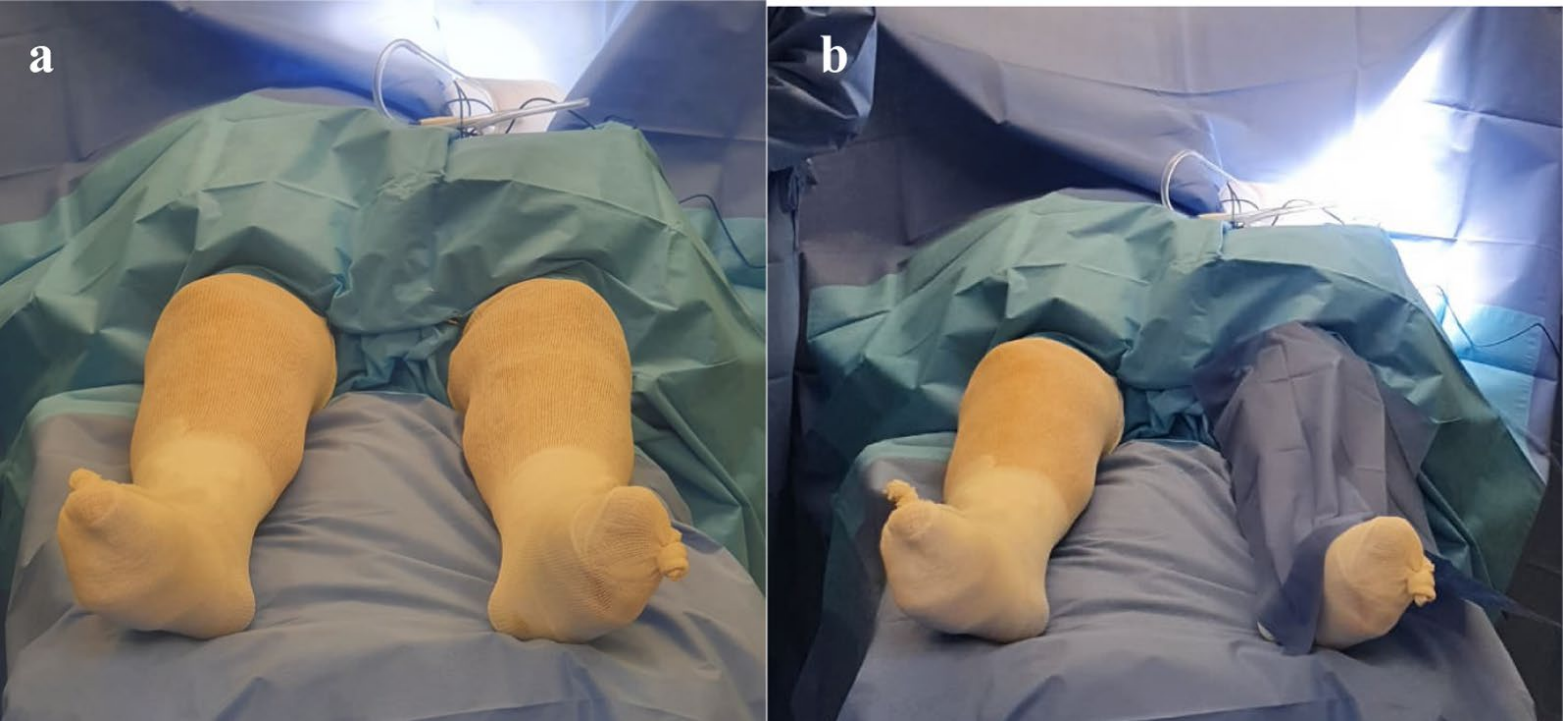
**a** Surgical field preparation of both lower limbs set for approach. **b** Left knee covered before approaching right knee

All patients were operated on a vertical laminar flow enclosure under spinal anesthesia with induced hypotension and without a tourniquet. We systematically administered one gram of cephazolin during anesthetic induction and two doses during the first 24 h after the procedure. We performed a full extension approach with a vertical midline incision and medial arthrotomy in both varus and valgus deformity, which were corrected with a standard soft tissue release prior to bone cuts (Fig. 1).

We carried out a generous resection of peripheral osteophytes, since no action was taken on the articular cartilage if the patella was retained, regardless of its macroscopical appearance. Although a bovie was used circumferentially to resect peripheric synovium, we do not acknowledge this action as performing a “patellar denervation” [22].

With the trial components in place, a thorough visual inspection is carried out to check for patellar height and tracking for the full range of motion. If any suggestion of maltracking was observed, a correction was attempted with a lateral release. If this was unsuccessful, we assessed the position and especially the rotation of both femoral and tibial components.

Closure of the extensor mechanism was always performed in full extension with multiple stitches of braided absorbable sutures. The skin was closed with separate nylon stitches at the beginning of the series and with a running intradermal suture. When the extensor mechanism is closed, and before skin closure, a full flexion test is again performed to evaluate tracking and eventual stitch loosening or breakage. All the components were fixed with bone cement, and subcutaneous low molecular weight heparin was administered 30 days after surgery as thromboprophylaxis (patients were trained to self-administer the rest of the applications at hospital discharge).

All the prosthesis used were posterior stabilized: 26 titanium tibial base PFC Sigma (Johnson & Johnson; Depuy, War. Ind. USA); 24 were all-polyethylene tibial base PFC Sigma (Allpoly® Johnson & Johnson; Depuy, Warsaw. Ind. USA); 20 were Scorpio® (Stryker; NY, USA), 10 United OC (Hsinchu, Taiwan), 2 Nexgen (Zimmer, Warsaw, Indiana, USA), 2 Optetrak (Exactech, Gainesville, Fla) and 2 Insall-Burstein (Zimmer, Warsaw, Indiana, USA).

Rehabilitation protocol was the same for all knees, with complete weight bearing and walker-assisted ambulation on the first postoperative day and progression to elbow crutches as tolerated. After the third postoperative week, minimal or no assistance was allowed. Active full extension was encouraged from the immediate postoperative hours, while flexion was encouraged but not forced. If the knee did not reach 90°of flexion at 3 weeks, the patient was sent to intensive physical therapy and was reevaluated at 6 weeks. If 90° were not achieved, manipulation under anesthesia was planned before the eighth week.

### Clinical and functional assessment

Preoperative and end-of-study of Knee Society Score (KSS) values [23] (with clinical and functional subscales) were registered. Subjective analysis for pain was performed with a Visual Analogue Scale (VAS) [24]. All the forms were completed by a fellow trained in knee reconstruction surgery during routine visits, and the comparison between the preoperative values and final follow-up was performed.

**Fig. 2:**
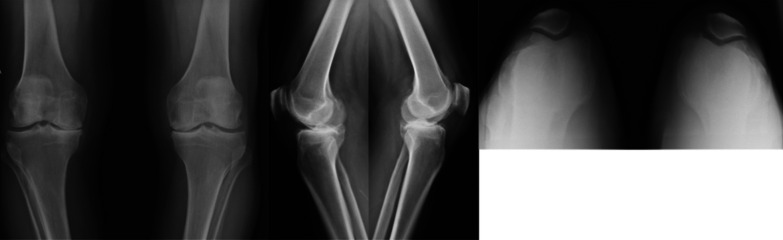
Preoperative AP, lateral, and axial views of a 63-years-old male patient, with bilateral genu varus

**Fig. 3.**
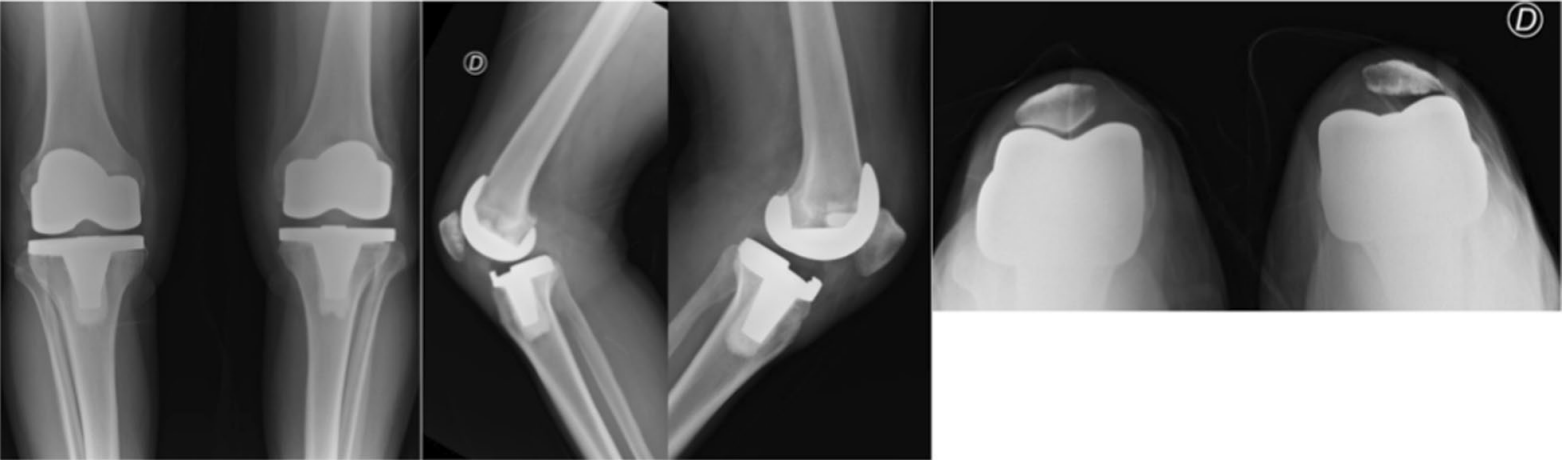
7.2 years of follow-up images. The right knee had patellar resurfacing and the left knee with patellar retention. No signs of loosening can be observed

Antero-posterior (AP) (with 10º degrees of lower limb internal rotation), lateral, and patellar axial radiographic views were used for analysis. Images were performed systematically before surgery and immediately after the procedure. Then, they were performed at 30 days, twelve months, and annually. Radiographic assessment was performed twice using Synapse software (Fujifilm, Medical System, USA) to reduce precision bias (Figs. 2,3).

The preoperative osteoarthritis stage was assessed using the Kellgren-Lawrence Scale [25]. The femorotibial axis was registered before and after surgery, and the patients were classified into three categories: normal values were considered between 5 and 7º of valgus [26]. Values above and below this were categorized as Genu valgus and Genu varus, respectively. Patellar height was assessed using the Caton-Deschamps method [27] in lateral projections with 30º degrees of knee flexion.

Mechanical loosening was defined according to the Knee Society system [28], and infection was considered according to the Musculoskeletal Infection Society (MSIS) and, recently, the World’s Association against Infection in Orthopaedics and Trauma (WAIOT) criteria [29, 30].

### Complications and revision rates

We registered all complications during and after surgery, such as infection, periprosthetic fractures (PPF), loosening, patellar clunk, maltracking, AKP, avascular necrosis, thrombosis, and death. We compared the data between the knees with patellar resurfacing and non-resurfacing.

### Statistical analysis

We described continuous variables as mean and standard deviation or median and interquartile range according to normality while categorical variables were described as frequency and percentages. Group comparisons were performed using the Mann–Whitney or Student test. Chi-square or Fischer’s exact method was used for categorical variables. All information was put into an Excel® (Redmon, USA) spreadsheet, and statistical calculations were carried out using GraphPad Prism® (Lajolla, CA, USA). A difference of *p* < 0.05 was considered statistically significant.

## Results

### Clinical and functional assessment

We observed statistically significant improvement regarding postoperative values of KSS, VAS and extension (*p* < 0.01) in both groups (Table 2). No differences were found at the end of the study when comparing the patellar resurfaced group versus the non-resurfaced group. Regarding Clinical KSS, we observed postoperative scores of 82.8 ± 7.4 and 83.2 ± 3.4 (*p* = 0.82) in resurfaced and non-resurfaced groups. Assessment of Functional KSS showed values of 89.1 ± 8.2 versus 90.4 ± 6.8 (*p* = 0.99) (Table 3). Summary of the results are described in Tables 2 and 3.

**Table 2:** Summary of preoperative and postoperative clinical and functional scales

Variables	Preoperative	Postoperative	*p* value
Resurfaced			
KSS _(mean, SD)_			
Clinical	44.6 ± 4.8	82..8 ± 7.4	< 0.01
Functional	45.3 ± 7.1	89.1 ± 8.2	< 0.01
VAS _(mean, SD)_	8.4 ± 0.9	2.0 ± 0.9	< 0.01
Caton-Deschamps _(mean, SD)_	0.9 ± 0.2	1.0 ± 0.4	0.84
ROM _(mean, SD)_			
Flexion	99.4 ± 12.9	108 ± 7.5	0.27
Extension	8.2 ± 4.1	3.2 ± 1.6	< 0.01
Non-resurfaced			
KSS _(mean, SD)_			
Clinical	45.5 ± 3.6	83.2 ± 3.4	< 0.01
Functional	43.2 ± 7.8	90.4 ± 6.8	< 0.01
VAS _(mean, SD)_	8.2 ± 1.0	1.8 ± 1.0	< 0.01
Caton-Deschamps _(mean, SD)_	1.0 ± 0.2	1.1 ± 0.1	0.77
ROM _(mean, SD)_			
Flexion	101.9 ± 9.1	110.3 ± 7.7	0.34
Extension	8.7 ± 5.2	2.8 ± 1.3	< 0.01

KSS: Knee Society Score. VAS: visual analogue scale pain. ROM: range of motion. SD: standard deviation

**Table 3:** Comparative assessment between both cohorts at the end of the study

Variables	Resurfaced	Non-resurfaced	*p* value
KSS _(mean, SD)_			
Clinical	82.8 ± 7.4	83.2 ± 3.4	0.92
Functional	89.1 ± 8.2	90.4 ± 6.8	0.99
VAS _(mean, SD)_	2.0 ± 0.9	1.8 ± 1.0	0.84
Caton-Deschamps _(mean, SD)_	1.0 ± 0.4	1.1 ± 0.1	
ROM _(mean, SD)_			
Flexion	108 ± 7.5	110.3 ± 7.7	0.56
Extension	3.2 ± 1.6	2.8 ± 1.3	0.67

KSS: Knee Society Score. VAS: visual analogue scale pain. ROM: range of motion. SD: standard deviation

### Complication and revision rates

The complication rate was 18.6% (n = 16); nine (10.5%) were observed in the resurfaced group, while seven (8.1%) were in the non-resurfaced group (*p* = 0.57). There were two (2.3%) cases of acute periprosthetic joint infection (one from the resurfaced and the other from the non-resurfaced group): one required surgical debridement and irrigation with the exchange of the polyethylene insert, and the other patient underwent a two-stage revision. Both patients did not report any recurrence at the end of the study. One patient (2.3%) of the non-resurfaced group developed deep vein thrombosis (DVT) that required specific medical treatment and achieved full recovery.

Thirteen (15.1%) patients reported AKP; eight (18.6%) were from the resurfaced group, and five (11.6%) were from non-resurfaced (*p* = 0.81).

We didn’t observe any cases of PPF, loosening, maltracking, avascular necrosis or death until the end of the study. Four (2.6%) lateral releases (two in each group) were necessary to achieve correct patellar tracking (*p* = 0.99).

## Discussion

The main finding of this study is that after an average of 7.6 years of follow-up, there were no significant differences in the knees with or without a resurfaced patella regarding AKP, patellar clunk, clinical and functional scores, complications, or revision rates.

The clinical impact of patellar resurfacing has been previously described, but no clear evidence exists that this should be performed systematically. In a prospective study, Agarwala et al. [6] reported 60 patients who underwent simultaneous bilateral TKA. Two comparison groups were recruited: those who had their patella replaced and those where a patelloplasty was performed. After a mean follow-up of 19 months, they did not observe statistically significant differences regarding clinical or functional scores. Grassi et al. [31] performed a systematic review and meta-analysis. They included ten studies assessing patellar resurfacing versus non-resurfacing; only two studies [11, 32] described better scores regarding KSS in favor of resurfacing. They argue that their findings demonstrate no clear superiority in performing patellar replacement and that these results should be interpreted cautiously due to the significant heterogeneity of the studies.

At the end of this study, after seven years of follow-up, statistically significant improvement was observed in both cohorts regarding KSS and VAS scores after surgery. However, no significant differences were observed when comparing the outcomes between the groups. We consider that the fact that patellar resurfacing and non-resurfacing were performed in the same patient highlights the importance of these findings by reducing potential confounders such as type of patient or pain perception.

Several authors have studied anterior knee pain after TKA. Although the literature shows a tendency to be more frequent in patients without patellar resurfacing, there is still no clear consensus [33, 34]. Wood et al. [35] conducted a prospective, double-blind, randomized trial, where they described that in patients where the patella was preserved, anterior knee pain rates were significantly higher than in patients with patellar resurfacing. However, this condition was also observed in the second group, and 10% (nine patients) of these patients required revision involving the patellofemoral joint. A systematic review and meta-analysis performed by Nizard et al. [11], twelve randomized studies comparing patellar resurfacing and non-resurfacing, found that the relative risk for AKP was 0.39 (95% CI 0.20–0.75; *p* = 0.005) in favor of resurfacing. However, when the analysis was limited to the three best-quality studies, this difference was no more significant between both cohorts. We believe that the similar rates of AKP observed in both groups in this study (18.6% vs. 11.6%; *p* = 0.81) suggest that not all AKP are due to patellar retention and that we should look for other causes in our examination: synovial plicae, component malrotation, tendonitis, patellar maltracking, although beyond the scope of this study, are well-recognized causes of knee pain that we should keep in mind for future research.

There is a tendency to believe that patellar retention during total joint replacement is associated with higher long-term revision rates [36, 37]. However, our results are contrary to those of these authors, as we have not registered any revision of the patellofemoral joint after seven years. There is a possibility that orthopedic surgeons are more likely to perform secondary patellar resurfacing surgery in patients with persistent pain and patellar retention, and this increases reoperation rates in the literature.

The clinical implications of our results are consistent with the fact that there is still insufficient evidence to demonstrate that one strategy is significantly superior to the other.

Our study has limitations. The absence of radiographic analysis of osteoarthritis progression or patellar thickness could increase the possibility of Type I error. In addition, using seven different prosthetic models could have affected the interpretation of the results.

Most studies discuss patellar resurfacing versus retaining on staged surgeries among the same or different patients. The fact that in this series, we included 43 patients undergoing one-stage bilateral TKA, where in one knee, the patella was replaced, and the other knee was retained, provides greater relevance to our findings. Continued follow-up is pending to assess long-term follow-up.

## Conclusion

Our findings suggests that in the contest of a TKA, patellar resurfacing did not demonstrate statistically significant differences concerning patellar non-resurfacing in clinical nor functional outcomes, AKP, complications, or revision rates after minimum 7 years of follow-up.

## Data Availability

The datasets used and/or analyzed during the current study are available from the corresponding author on reasonable request.
